# Supplementary material for: Cover versions as an impact indicator in popular music: A quantitative network analysis
Source: PLoS One. 2021 Apr 19;16(4):e0250212. doi: 10.1371/journal.pone.0250212 (PMC8055012; doi:10.1371/journal.pone.0250212)
Supplement: S2 File — (DOCX) [file pone.0250212.s002.docx]

Normalization of covers by the number of released songs

| Name | Songs | Starting decade | Genre | weighted indegree | normalized weighted indegree |
| --- | --- | --- | --- | --- | --- |
| Franz Gruber | 2 | 1890s | Classical | 3157 | 1578.5 |
| Joseph Mohr | 2 | 1890s | Classical | 3157 | 1578.5 |
| Emily Laurey | 1 | 1890s | Classical | 1557 | 1557 |
| Meister Glee Singers | 1 | 1890s | Vocal | 983 | 983 |
| Hollace Shaw | 1 | 1930s | Stage & Screen | 975 | 975 |
| Hiram Sherman | 1 | 1930s | Vocal | 966 | 966 |
| Gloria Grafton | 1 | 1930s | Stage & Screen | 944 | 944 |
| Tally-Ho! | 1 | 1900s | Vocal | 919 | 919 |
| Kathryn Crawford | 1 | 1930s | Stage & Screen | 770 | 770 |

Table 5. Top 10 artists according to the normalized weighted indegree by the number of released songs

Table 5 presents the result of normalizing covers by the total number of released songs. Table clearly shows that these artists have a very high impact due to only one or two songs. Franz Gruber and Joseph Mohr are the authors of the famous Christmas carol “Stille Nacht, heilige Nacht” and Emily Laurey premiered the carol “Minuit, chrétiens/O Holy Night”. The other ones are one-hit wonders with only a punctual popular hit. This result illustrates that the normalization would produce artefacts that favor the impact of artists with low production and the occasional huge impact of one song. As it can be seen, this normalization could be more appropriate to value the impact of specific songs, than the complete career of artists.
