# Supplementary figures and images for: Cover versions as an impact indicator in popular music: A quantitative network analysis
Source: PLoS One. 2021 Apr 19;16(4):e0250212. doi: 10.1371/journal.pone.0250212 (PMC8055012; doi:10.1371/journal.pone.0250212)

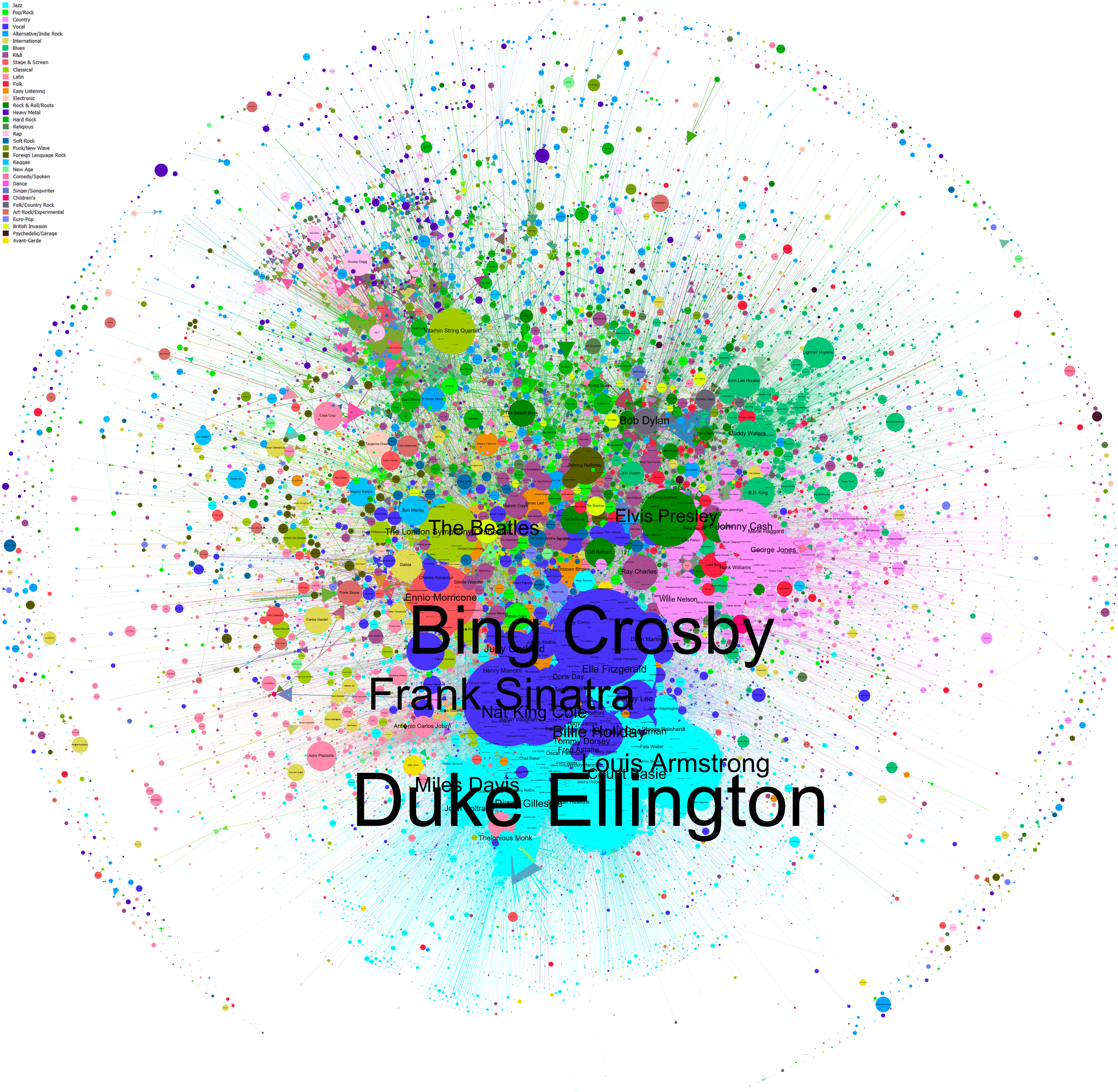

Supplement: S1 Fig — (TIF) [file pone.0250212.s003.tif]
