# Supplementary material for: Cover versions as an impact indicator in popular music: A quantitative network analysis
Source: PLoS One. 2021 Apr 19;16(4):e0250212. doi: 10.1371/journal.pone.0250212 (PMC8055012; doi:10.1371/journal.pone.0250212)
Supplement: Attachment — Submitted filename: Reviewers_response.docx [file pone.0250212.s004.docx]

Reviewers' comments:

Reviewer's Responses to Questions

**Comments to the Author**

1. Is the manuscript technically sound, and do the data support the conclusions?

The manuscript must describe a technically sound piece of scientific research with data that supports the conclusions. Experiments must have been conducted rigorously, with appropriate controls, replication, and sample sizes. The conclusions must be drawn appropriately based on the data presented.

Reviewer #1: Partly

Reviewer #2: Yes

2. Has the statistical analysis been performed appropriately and rigorously?

Reviewer #1: No

Reviewer #2: Yes

3. Have the authors made all data underlying the findings in their manuscript fully available?

The [PLOS Data policy](http://www.plosone.org/static/policies.action#sharing) requires authors to make all data underlying the findings described in their manuscript fully available without restriction, with rare exception (please refer to the Data Availability Statement in the manuscript PDF file). The data should be provided as part of the manuscript or its supporting information, or deposited to a public repository. For example, in addition to summary statistics, the data points behind means, medians and variance measures should be available. If there are restrictions on publicly sharing data—e.g. participant privacy or use of data from a third party—those must be specified.

Reviewer #1: Yes

Reviewer #2: No

4. Is the manuscript presented in an intelligible fashion and written in standard English?

PLOS ONE does not copyedit accepted manuscripts, so the language in submitted articles must be clear, correct, and unambiguous. Any typographical or grammatical errors should be corrected at revision, so please note any specific errors here.

Reviewer #1: Yes

Reviewer #2: Yes

5. Review Comments to the Author

Please use the space provided to explain your answers to the questions above. You may also include additional comments for the author, including concerns about dual publication, research ethics, or publication ethics. (Please upload your review as an attachment if it exceeds 20,000 characters)

**First of all, I would like to thank to the reviewers their comments because their suggestions have led me to detect important gaps in the manuscript and explore the evolutionary aspect of the data with more detail. Great part of the manuscript was then rewritten to centre the manuscript in how the network has evolved in successive decades.**

Reviewer #1: The manuscript “Cover versions as an impact indicator in popular music: A quantitative network analysis” uses a large crowdsourced database of musical covers (the performance of an already existing song by a different artist) to look at the network of covers. The analysis extracts the most central artists according to three measures of centrality. It also examines the connections between genres of covers. Finally, it provides some temporal analysis of how covers have evolved over the last century.

I think that the question of how covers result in social and musical connectivity is interesting and that networks are an appropriate method for parsing this phenomenon. However, I am not sure that the analysis in the manuscript has used networks to their fullest extent, and many of the analyses seem to be confounded by time. The question of time, as in how long an artist has existed, seems to be central to many of the analyses---it is the motivation for introducing PageRank, and at the center of many of the genre analyses. However, for how much it comes up, I do not think it has been directly and adequately addressed.

For example, the analysis observes that ranking artists by in-degree results in a number of older bands. It then states “a more precise way to reduce the oldness effect on the musical impact is by calculating its PageRank.” I do not think that PageRank is a sufficient answer here---it does explicitly not do anything to guarantee that time is properly accounted for in the analysis. A number of the artists in Table 2 are still from the 1920s and 1930s (brief aside: I would like to see more than the top 10 artists, perhaps as an appendix table). Instead, I think time should be tackled directly: we are interested in who the most central artists are in the cover network, but an artist is more likely to be covered simply if they are around longer. A simple analysis might be normalizing the in-degree by the number of years since the artist first became active. A more comprehensive analysis, that I would like to see, would treat the cover network as a temporal network and use temporal centrality measures to understand how artists have become more or less central over time. The individual artists section culminates in the statement that “musical oldness of artists is a key factor to improving the likelihood of being covered.” This seems more like the analysis hasn’t properly accounted for time, rather than a result. That is why I would like to see time handled more directly.

**Section “Degree distributions” has been rename and broaden as “General characteristics of the covers network”. Now, the global network has been split by decade in which the artists start their music career, covering since 1910s to 2010s. This allows us to study how the network has evolved as new artist were aggregated. Apart from the indegee and outdegree distributions (Figure 1), this section now presents several global network indicators (Figure 2 and Table 1) such as Weighted average degree and Clustering coefficient that describe the characteristics of the network in each decade. This has allowed to observe different growing patterns. In addition, Figure 3 (former Figure 6) has been added to explain the possible causes of the irregular growth of the network.**

**A table with the network indicators of the artists and a large-size version of Fig 6 was added as supplementary material.**

The genre analysis does a bit more to look at temporal dynamics, but it also avoids probing too deeply. I think Figure 6 is interesting. I think that it would work better as a heatmap where the rows and columns are years, and the color is Figure 6’s y-axis. I would like to know more about what is going on here. What genres are driving these temporal lags in covers? How does the centrality of genre covers change as a result in these lags?

**Figure 6 (Now Figure 3) has been redone as a heatmap. Now, differences between generations are better appreciated. Section about genres has been remade to emphasize how the preferences covering genres have changed (Figure 8). This result attempts to explain the structural changes observed in the growth of the global network.**

I think that the manuscript struggles to pinpoint particular temporal questions to answer because its research questions are relatively broad and the main motivation is to do a quantitative analysis. I do not think doing a quantitative analysis necessarily tells us anything new, and so I don’t find the “total absence of quantitative studies” to be that compelling (though isn’t that also contradicted by the previous two paragraphs in literature review?). However, the manuscript mentions that qualitative studies have led to theorizing about the cultural contribution of covers (references 20-22). I think there is a missed opportunity here to use the large-scale network analysis to help clarify and expand upon what has been noticed in the theoretical/qualitative studies. I think by engaging more closely with the prior literature and synthesizing it into hypotheses that have not been tested would help generate a more productive analysis, and help guide questions about how time affects cover centrality.

**The objectives of the paper have been changed due to the new approach of the manuscript and all the objectives include now an evolutionary perspective.**

Overall, I would like to see the issue of time addressed more clearly and more directly in the analysis. I think it confounds how we interpret both the individual and genre analyses. To this end and deriving questions related to how time affects the cover network, I think the manuscript would benefit from engaging with the hypotheses of the prior literature, and building from them, rather than doing a general descriptive analysis of the network.

**Results section has been enriched with longitudinal analyses that respond to the research questions. Now, the discussion about these results has been improved adding more previous studies that support the interpretations**

Other comments
- The introduction should tell me what the questions are and how the analysis proposes to answer them. It ends abruptly before going to the related research. Similarly, the manuscript says that the objective is “to visualize and analyze the cover songs network between artists in order to describe its topology and identify the main nodes.” But why do that? What do we learn from making a network and identifying the main nodes that we didn’t know before? The final conclusion is “These results further strengthen the quantitative approach in studies on the history of music.” But what do they strengthen? What are they speaking to?

**A paragraph was added at the end of the introduction to define the principal purpose of the paper. As I have commented, objectives have been changed and now more accurate research queries were formulated. Conclusions section was added to give more relevance to this part and new conclusions were written to precise the main findings.**

- I am worried about duplications in the data. In data cleaning, it is mentioned that if an artist covers the same song more than once, it is counted as a different cover. Has there been any validation to ensure that those covers of the same song are not exactly the same cover?

**This only occurs with songs that share the same title. It is very infrequent that an artist covers a same song from the same artist in different moments. In spite of this, I think that each cover versions shall be counted as different because it is created in a different context and they express the strength in the connection between artists.**

similarly, I am worried that the number of songs in the Tables are extremely high. Duke Ellington was prolific, but I think it is unlikely that he wrote (or performed?) 7,447 songs. Similarly for the other artists, though there is then a huge gap with Franz Gruber and Joseph Mohr. I would like ot know what is going on here better. Why are the numbers so large?

**The number of songs are obtained from Allmusic.org page. This site gathers all the songs recorded by an artist. This link shows the current number of Duke Ellington songs, 7621.**

<https://www.allmusic.com/artist/duke-ellington-mn0000120323/songs/all>

**This amount is only used to express the size of the node in the graph and they are used to illustrate the production of musicians. They are not relevant in the analysis.**

**However, in my opinion that volume of recorded songs is common in important music starts. For example, Bing Crosby has close to 9000 songs**

[**https://www.allmusic.com/artist/bing-crosby-mn0000094252/songs/all**](https://www.allmusic.com/artist/bing-crosby-mn0000094252/songs/all)

**The Beatles, 2000 songs**

<https://www.allmusic.com/artist/the-beatles-mn0000754032/songs/all>

- These analyses do not establish that the cover network is scale free and I think that the language of “scale free” should be left out of the paper. The degree distributions do not look scale free to me visually, and no test has been reported to indicate that they follow a power law. What is alpha? Relatedly, the discussion of “preferential attachment” should be dropped because no analysis establishes that there is preferential attachment in the network (exactly because there are no analyses that directly model the time of artists and receiving covers). I think that an analysis of preferential attachment would be interesting, and it can be done (for example, see Laszlo Barabasi’s textbook for measuring preferential attachment), but it is not done here. Even if it is done, I think the language of “scale free” should not be included---preferential attachment in a real world system does not guarantee a power law degree distribution.

**Allusions to scale free have been avoided. Due to the new perspective of the paper, focused on the evolutionary aspects, any issue on the type of network is irrelevant and it has been removed.**
**Alpha is the exponent of the curve. This has been included in the text (p. 8)**

- How PageRank is framed through the paper is inconsistent. In the introduction and abstract, it is described as measuring the “most influential.” In the analysis, it is described as accounting for time (which I don’t think it does). The intuitive description of PageRank when it is introduced as a metric is correct, and that should be used to describe in the other parts of the paper.

**That reference about the use of Page Rank for counteracting time effects is wrong and was removed. This metrics is now only used as influence indicator.**

- I don’t believe there is a sentence that explicitly says what the nodes and edges are in the network. Specifically, I want to know if the nodes are artists from SecondHandSongs, or from Allmusic.

**This sentence was added to *General characteristics of the covers network* in p. 8: *“A network graph was constructed with 106,096 nodes (artists) and 855,650 arcs (cover versions) extracted from SecondHandSongs”***

- In the SecondHandSongs dataset, what does it mean for a song to be labelled as “traditional.” In the Allmusic dataset, what is meant by “genres and styles are not interconnected”? Isn’t a network made of them later?

**Traditional means that the first performer of the song is unknown, so then it is not possible to create a link between covered artist and subsequent performers.**

**That sentence about the interconnection of genres and styles is a mistake and that idea is wrongly expressed**. **A new text was added: “*Styles are not considered as sub-categories of genres, then they are used as labels or keywords that are freely assigned to an artist.*”**

- The SecondHandSongs dataset section first says that there are 96 thousand songs. The data extraction paragraph says that 155 thousand songs were extracted. Why are they not the same number?

**There is a mistake in the count of original songs. The sample only include titles of songs that have been covered, so 155k is actually the list of titles included in the sample, both covers and original songs. I have decided to remove that figure because it would be confused.**

- Details about song and artist IDs do not need to be included in the manuscript.

**I think that this information is very important to reproduce the sample. This detail about the IDs allows other researchers to extract data from SecondHandSongs and Allmusic.**

- I do not understand the inclusion criteria for the genre network: “Only the most important influences were highlighted through percentages of covers equal to or higher than 10% the total number of covers made.”

**This is a pruning process that selects only the most important links. The reason is that all the genres have links among them, resulting a ball-shape network. This pruning criterion ensures that only the most significant relationships are drawn in the picture, producing a more interesting graph. This text was added in p. 18: “*The graph only includes links that are equal or higher than 10% of all the cover songs of other genres (outdegree). For example, Jazz adapts 40% of Jazz pieces and 24% of Vocal songs and is an inspiration for 65% of Jazz and 10% of Vocal songs. This pruning criterion allows to select only the most important relationships among genres.*”**

- The names of artists and bands should not be italicized.

**Corrected**

Reviewer #2: The manuscript ‘Cover versions as an impact indicator in popular music: a quantitative network analysis’ deals with the analysis of a database of cover songs through the lens of networks, where two artists are linked if one covers a song of the other. The resulting network is then used to measure the influence between artists and to exploit the connections between their respective genres.

The idea is (to the best of my knowledge) new, the results are interesting, and the presentation clear (the figures not so much). Overall, I believe that the manuscript deserves publication in PLoS One, but there are some points that I would like the authors to discuss first:

1) As far as I understand the network representation adopted by the authors, there is a directed link between artist i and artist j if i covers a song of j. The authors also write "Links between artists are therefore established according to the number of times an artist covers another", so this makes me think that links are weighted. However, all the measures used do not take weights into account. This is an important point that needs to be clarified. In case the measures take weights into account this should be explained better. Otherwise, why not considering weighted measures (in-/out-strength instead of degree, betweenness centrality defined on the shortest paths that consider weights etc.)?

**I agree with you, and it was a mistake to use those metrics for a weighted network. Degree was replaced by the Weighted degree. Now, the results are more consistent.**

2) Is there a way to account for the size of the production of different artists to make the cover flows comparable? For example, an artist with a huge production that gets covered a lot would could in principle be comparable with an artist with a smaller production that gets covered a bit less. The same argument holds for genre-to-genre comparisons, since the production in each year and for each genre can change. I believe that counters should be somehow normalised to be comparable. See for example what is usually done for citation networks [Radicchi, F., & Castellano, C. (2011). Rescaling citations of publications in physics. Physical Review E, 83(4), 046116.] or [Waltman, Ludo. "A review of the literature on citation impact indicators." Journal of informetrics 10.2 (2016): 365-391.] to assess the influence of authors and temporal flows of knowledge across different fields [Sun, Y., Latora, V. The evolution of knowledge within and across fields in modern physics. Sci Rep 10, 12097 (2020)].

**This is an interesting point because one could assume that some type of normalization is necessary to avoid size-effects. However, this issue is problematic from a conceptual perspective. Firstly, we are talking about musicians, not about scientists. The concept of production (number of papers vs. number of songs) and impact (number of citations and number of covered songs) cannot be literally translated. In Science, any paper includes bibliographic references (it is almost impossible to publish a research article without references), then as more articles more citations. In other words, citations are dependent of the publications.**

**However, songs do not include any reference to previous releases, musicians are not determined to cover songs of other artists. Therefore, more songs do not imply more cover versions. In fact, there are musicians with a large trajectory and very low number of cover versions, and the contrary (e.g. tribute bands). This is confirmed by the low correlation between the number of songs recorded and covers done (weighted outdegree) (r=.465) and received (weighted indegree) (r=.443). Then, the use of a normalized count would cause numerical artefacts, undervaluing great musical figures with an extensive career and large production (e.g. Duke Ellington, Bing Crosby) and overvaluing unknown artists whose exclusive merit is to firstly interpret a popular song such as *Silent night* by Franz Gruber and Joseph Mohr, or *Summertime* by Abbie Mitchell**. **In my opinion, the use of normalized counts would cause confusing results.**

Below a list of minor points:

1) Abstract: the sentence "However, no studies have explored the meaning of covers from a quantitative and structural perspective" sounds a bit vague to me. What is the meaning of a cover? I would suggest rephrasing it. (the paper is not about the meaning of covers as far as I see it). Also, the structural perspective might be misleading here, since it's not on the structure of the cover, but covers are used to create a structure between artists.

**Abstract was rewritten and that sentence was removed due to its ambiguity. Now, the abstract is focused on the new results and their conclusions.**

2) Introduction: "These acknowledgements lead to (...) while assessing the impact of artists’ careers". As this sentence is written at the moment is seems that covers access the impact of the careers. It should be rephrased.

**The sentence was rewritten: “*These acknowledgements lead to links between artists and illustrate the musical influences of soloists or bands, while enabling to assess the impact of artists on specific musical communities.*”**

3) Introduction, last sentence: "who their songs were versioned by" sounds a bit weird.

**The sentence has been rewritten: “…*including information about cover songs and their performers.*”**

4) Related research, third paragraph: "Nevertheless, an important gap in the analysis of cover songs is the total absence of quantitative studies." The second paragraph presents some quantitative studies on music, not on covers. Given that the third paragraph starts with 'nevertheless', I would swap the writing to emphasise the lack of analyses of covers among the quantitative studies rather than the lack of quantitative studies among the analyses of covers.

**I agree with your observation. The text was rephrased: “*Nevertheless, an important gap in the quantitative studies on the contemporary popular music is the total absence of analysis on cover versions. In most of the cases, they have been observed from a theoretical perspective, figuring out the cultural meaning of this type of songs.*”**

5) Objectives. The second point reads "Could social network measures be used to detect different types of impact?". I understand this is precisely what the manuscript is about, but I do not think that the results of the paper actually answer this question. The manuscript does not compare the ranking induced by the measures with some "ground truth" influence of artists, since it obviously does not exist (maybe some proxy for popularity could be used). Thus, since the authors are not testing the ability of the measures to capture the influence, but are proposing to use the measures as a proxy for influence, I would change/remove the question.

**That research question was reformulated: “*Which musical artists are the most influential in the network and how have they reached that position? What information does each network metric provide about the impact?*”**

6) A clear definition of the network representation and nomenclature is missing. This would help the reading of the following 'social network analysis' section, where nodes and lines are mentioned (also, why not calling them links?).

**This information is now included at the beginning of the *General characteristics of the covers network.***

7) Degree distribution section: the \alpha is not defined. Also, which method is used to fit the distribution? This should be specified. The fit does not seem to capture well the tail of the out degree distribution.

**This text was added to explain the power-law distributions: “*This interpretation could be confirmed by the cumulative distribution of covers performed (outdegree) and received (indegree) by artist. They follow a power-law distribution (Figure 1), with an exponent (α) of 2.882 for outdegree and 2.937 for indegree.*** ***R package poweRlaw was used to create the cumulative distribution functions (CDF) and the maximum likelihood estimation (MLE) method was used to fit the distributions.*”**

8) The authors focus on the Page Rank and the betweenness centrality, but there is obviously a long list of additional measures that could be used instead to produce rankings in -evolving- networks (Liao, Hao, et al. "Ranking in evolving complex networks." Physics Reports 689 (2017): 1-54.). For example, the alpha-centrality could be a good proxy for influence in static graphs. Why did the authors restricted their attention to the selected measures?

**The study is focused on three metrics: Weighted indegree, PageRank and Betweeeness centrality. These metrics provide three different points of view about the impact: Weighted indegree offers information about how the impact is accumulated, PageRank about the influence capacity of artists, and the betweenness centrality measures the ability to be covered by different and distant artists. These metrics were selected by several reasons: (1) they are well known by a generalist audience (PLOS ONE is a multidisciplinary journal), (2) their meaning and calculation is easy to understand, then it is easy to translate their meaning to a different environment such as the popular music, and (3) they are implemented in many network analysis software packages (Gephi, Pajek, VOSviewer, etc.).**

**I agree that there much more network metrics, but this is a study about cover songs and I think that three metrics is enough to have a good view about the impact of the musicians. I think that the inclusion of more metrics would not improve the results nor provide additional information. For example, alpha-centrality belongs to the eigenvector family (PageRank is a version of eigenvector for directed networks). It is very likely that the result would be similar than the observed with PageRank.**

9) Just before Genres and styles section: "This result confirms that the musical oldness of artists is a key factor to improving the likelihood of being covered". This makes sense, but isn't there already a bias towards the "old" artists that survived against the new ones? There would be "old" forgotten ones that did not even appear in the database, so oldness in this sense already encodes an inner survivorship bias. The authors could maybe expand on this.

**That paragraph was removed due to that result was obvious and to make way to other relevant results.**

10) Fig 3 is not very readable.

**Figure 3 (now Figure 6) was changed by a more complete figure. The problem of this figure is the size and resolution. I think that the journal would publish a large-size version of the figure that allows to explore the connections and groups.**

11) I really like the idea behind Fig. 4. Would it be possible to visualise it also with a heatmap instead of a network? It would make possible to directly compare flows within and across genres. Ideally, it would be interesting to see how these flow evolved in time and reorganise due to the appearance of new genres.

**Figure 8 was added to visualize the evolution of cover songs by music genre. This figure illustrates the preference by certain genre in each decade.**

12) Fig. 5 and Fig. 6 do not have labels.

**Both figures has been removed**

13) Fig. 6 is not easily to interpret, since lines typically denote an evolution, while here each point is a percentage. Also, I noticed something that I do not understand. As I understand the chart, there are songs from artists of the 90s that are covered by artists that started in the 1910...(the lightest blue peak at 1990). How long can a career be? This should be checked, and maybe a simple stacked bar chart would facilitate the visualisation? Otherwise, I would suggest to use a heatmap with the years of the two artists on the axes and the proportion of covers as color intensity. This would make the comparison easier.

**Figure 6 was change by Figure 3. I agree with you and I think that a heatmap is more suitable to represent that idea.**

**You comment about artists from the 1910s cover songs from 1990s artists. This is a small artefact caused by the low proportion of songs and artists from 1900s decade in the database. The reason of that weird result is that there are some old orchestras and choruses (e.g. The London Symphony Orchestra) that have a large trajectory and they sometimes cover songs from Pop/Rock stars.**

14) In the conclusions the authors state that "Most interestingly, perhaps, these influences are the result of cycles." The connection between influence and cycles is not clear to me and could be further explained.

**Conclusions were completely rewritten and that sentence was removed. The new analysis makes more clear the connection between cycles and influence.**

15) Here some related papers that might be of interest:
- Serrà J, Corral Á, Boguñá M, Haro M, Arcos JL (2012) Measuring the evolution of contemporary western popular music.Sci. Rep.2:521.
- Zivic PHR, Shifres F, Cecchi GA (2013) Perceptual basis of evolving western musical styles. Proc. Natl. Acad. Sci. U.S.A.110(24):10034–10038.
- Youngblood, M. (2019). Conformity bias in the cultural transmission of music sampling traditions. Royal Society open science, 6(9), 191149.

**Many thanks for the references. I have decided to cite the last reference (Youngblood, 2019) because this study analyses music samples (a bit close to the cover versions) and it is focused on the factors that influence their selection. The remaining ones have been discarded because they are centered on musicology aspects such as the sound, melody and rhythm.**
