# Supplementary material for: Cover versions as an impact indicator in popular music: A quantitative network analysis
Source: PLoS One. 2021 Apr 19;16(4):e0250212. doi: 10.1371/journal.pone.0250212 (PMC8055012; doi:10.1371/journal.pone.0250212)
Supplement: Attachment — Submitted filename: Response to Reviewers.docx [file pone.0250212.s005.docx]

Reviewer #1: The manuscript “Cover versions as an impact indicator in popular music: a quantitative network analysis” has improved with the revisions. I think that identifying the split in what decades are covered (1920s vs 1950s) is interesting, and I think it has helped create a more coherent story around the other analyses. I have some reservations and questions that I would still like to see addressed.

1. Table 1 and several of the figures rely on networks constructed across time. How exactly were those networks made? Is it a cumulative network, where, for example, any node and link in the 1910s network is also in the 1920s network, and any node and link in the 1910s or 1920s network is in the 1930s network? I think this is the proper way to construct the network for the analyses. But it’s not clear if instead these might be snapshot networks, where only nodes from a particular decade and who they cover are included. This should be explained in the text.

**I have introduced some lines to explain that the longitudinal analysis is done over cumulative networks:**

***“In order to study the evolution of the network, artists were classified by the decade in which they started their musical career. During this process, 4,983 artists were discarded because they did not have that information. Then, several cumulative networks by decade were built. This aggregate approach is because the cover versions are made of current or precedent artists and each network rests on the previous one.*”**

2. I am still skeptical of duplicates in the songs used to count the productivity of each artist, as seen in Table 2 for example. Looking even just at one artist, Duke Ellington, it was easy to see that several “different” songs were actually the same one. I suspect it is similar for the other artists. If this measure of productivity is used in the paper, it should do deduplication.

**The number of songs reported by Allmusic is not exactly the total number of different musical pieces interpreted or recorded. Rather, it is the total number of releases, including different recording of the same songs. Due to this problem, that metric is not used in the study as productivity measure and it is included only for illustrative purpose. Even then, I decided to include a footnote to explain the meaning of songs:**

**“*Data supplied by Allmusic. Number of musical tracks recorded and released by artist. It may include different recordings of the same song.*”**

Also, it is not clear how artists were matched across SecondHandSongs and Allmusic. Is it just if an artist’s name matches exactly across the two datasets? This should be explained.

**A paragraph was added to Data cleaning section explaining how the match was made:**

**“*The name of the artist or band was used to match SecondHandSongs and Allmusic records. This element was carefully cleaned to avoid mismatches between slight name variations. Thus, some characters such as ampersands and punctuation marks were removed or replaced by normal letters (Simon & Garfunkel by Simon and Garfunkel, “Big Mama” Thornton by Big Mama Thornton). In other cases, names with different variations were normalized (Booker T. and the Memphis Group’s by Booker T. and the M.G,’s, Johnny Burnette Trio by Johnny Burnette Rock and Roll Trio); synonymy between different artists (Tom Jones, 60s vocal artist and Tom Jones, 50s lyricist) was solved adding a digit to the name.*”**

3. I am still unclear on what nodes ultimately end up in the networks because there are several similar but conflicting numbers about the number of nodes
- In Data Sources, it says the site “reported 788k covers and, 143k artists”
- The paper reports that “143k artists and 1.1 million covers” were extracted. This contradicts the first number. Is it because the previous stats were reported at an earlier time? If so, I think they should be dropped because they are confusing and appear to contradict the data collection.

**You are right those figures are contradictory, the total number of covers reported by the site in January 2020 should not be less than the total retrieved covers. The problem is that the “1.1 million covers” refers to an unpolished data sample, which also included originals, samples and medleys. I have removed that text and I have added a new paragraph in Data cleaning explaining all the transformations of the sample and the final number of covers and artists used in the study:**

**“*When a song is traditional or the original performer is unknown, SecondHandSongs assigns it to a generic unknown (20,428). These covers were removed because there is not a connection between artists. In addition, SecondHandSongs joins the code of two or more artists when they perform together (e.g. Sinatra and Bono, Elvis Presley and the Jordanaires). These cases were duplicated, assigning the same song to each artist. After all these transformations the final sample included 106k artists and 855k cover songs.*”**

- IDs 0 to 175000 were extracted. Why 175000 if there are only 143k artists?

**SecondhandSongs and Allmusic use sequential IDs. This has the problem that when a record is removed (i.e. duplicate, wrong name, etc.), the gap is not filled. Then, the total number of records in the database is always less than the last IDs. In addition, and due to this problem, it is necessary a large IDs edge, to be sure that there are no more records after some empty IDs.**

- The network is reported to have 106,095 nodes. Why is it not 143k?

**This is explained in the new paragraph in Data cleaning.**

- The last column of Table 1 reports that there are 101,112 nodes in the 2010s network. Why is it not 106,095?

**This is due to 4,983 artists do not have starting period date. The reason is that they are not included in Allmusic or it does not provide that information for those artists. This line was added to the second paragraph of *General characteristics of the covers network* section:**

***“During this process, 4,983 artists were discarded because they did not have that information.”***

If there are data processing steps that are causing these different numbers, they should be clearly reported next to each step.

4. Figures 4 and 5 help make some of the findings clear. It is difficult to tell which curve is associated with each artist. I would suggest placing each artist’s name at the end of each curve, so that they are all aligned on the right side of the plot.

**I have decided to insert a fine line to connect each label with the trend. The idea of putting the label at the end is fine, but the problem is that some lines finish very close among them and there is no place for the labels.**

Figure 8 is interesting, but it is very difficult to tell which color lines up with which curve. I think a heatmap-style figure like Figure 3 would be more clear.

**Lines of the Figure 8 has been replaced by a heat map.**

5. There are several places in the paper where it says it will do an analysis that is more than what is actually done
- In Top Covered Artists, the paper says “This section analyzes who are these figures and how they have acquired their positions.” The analysis does not say anything about *how* they acquired their positions in the network, just their importance in it.

**The sentence has been rewritten: “*This section analyses who are these figures and how they have evolved.*”**

- In Top Covered Artists, the paper says “In general, as the cover network evolves over time, the impact of new figures is greater and faster.” This has not been shown in general.

**The sentence has been rewritten: “*In general, this result suggests that as the cover network evolves over time, the impact of new figures is greater and faster.”***

In the following sentence, it also hasn’t been shown that anything is specifically “exponential.”

**The sentence has been rewritten: “*This would cause that the covering of songs is more and more concentrated in few artists, increasing extremely their impact over time.*”**

- In the Discussion, the paper says “Regardless of these limitations, the results could be representative of mainstream popular music.” That this is representative is not clear to me, I don’t think the paper needs to say this.

**That sentence was removed and replaced by: “*These limitations can influence the results relative to the relationship among genders.*”**

I particularly would like to see the methods questions answered. Addressing the other points would help make the paper’s story more clear and reliable.

-------

Other comments:
- The paper’s language switches tenses often. Sometimes it’s past tense, sometimes it’s present, sometimes it’s conditional. A reliable tense should be used throughout the paper.

**Paper has been revised to uniform the tenses**

- Network metrics (“weighted in-degree”, “clustering coefficient”) should not be capitalized throughout the paper.

**Corrected**

- The abstract is jarring: it jumps right into explaining data after barely motivating why the structure of covers is interesting (and it is interesting).

**It is possible that the abstract quickly focuses on methods and results, but there a 250 words limitation and I think that it is more relevant to summarize the results than including sentences about the motivations of the study.**

- The density by itself does not tell us much about the centralization of the network. Many empirical networks have very low density. What is more interesting is the density over time. When the density is reported, I think the “less than” sign is backwards, it should be (d < 0.01%) if I understand correctly. Also is it that d < 0.01% or 0.01? A density of 0.0001 for 0.01% does seem very low.

**It was a typo error. Because density is the proportion of links over the total possible links, then it is common to be expressed in percentages.**

- It still wasn’t clear to me immediately how the edges of the genre network were selected. If I understand correctly, it’s more clear to say something like “The edge weights were normalized per each node, and any weight below 0.1 was removed. This is the same as only including links where at least 10%...” For future reference, this is similar to but less statistically rigorous than using a backboning method like the disparity filter.

**Many thanks for the suggested text. I have included it in the paper. I used this criterion to prone the network because I did not find other way to do it. Thank you for your recommendation, I see that disparity filter has a R package, so I will try to use it in next studies.**

- In the discussion, it’s not clear if the “deep transformations in the way of making music” are speculations, or already known by previous work. How can “subsequent performers from the 1960s quickly overtake the number of covers of the previous generations” if “the cover song lost prominence because musicians compete now”?

**The exact meaning of the sentence is a speculation mine but based on the previous references. In general, academic literature agrees that the history of contemporary popular music in the 20^th^ century is defined by two differentiate epochs: Jazz and Rock.**

**This sentence was added to explain those contradictory statements: “*Taking into account that the covering of songs descends since 1950s, the fast success of these figures could illustrate an extreme concentration of the impact in special artists thereafter.*”**
-------

Typos and phrasing (since PLoS ONE doesn’t provide copy editing):
- Page 1, “network metrics have allowed to identify” is missing a word I think
- Page 2, “while enabling to assess the impact” is awkwardly phrased

**The sentence was rewritten: “*while assessing the impact of artists’ careers in specific musical communities.*”**

- Page 4, “In this form, it attempts to know how the use of” is awkward with “it attempts to know.” The paper can be more definitive.

**The sentence was rewritten: “*it attempts to understand how*”**

- Page 5, In the third research question, “possible to appreciate” is awkward since “appreciate” is usually meant in terms of subjective terms.

***Appreciate* has been replaced by *observe***

- Page 5, “the database has been fed,” “fed” is an awkward verb. Databases don’t eat

***Fed* has been replaced by *supplied***

- Page 5, “the song is labeled as traditional,” put quotes around “traditional” to make it more clear it’s a label, and say that it’s labeled that way by the dataset (I believe?) rather than by the researcher

**That sentence was removed because it is better explained in Page 7**

- Page 6, “Styles are not considered as subcategories of genres, then they are used as labels”, something isn’t right with the second half, “then they are used as”

**The sentence was rewritten: “*therefore, they are labels or keywords freely assigned to an artist.*”**

- Be consistent with “arcs,” “lines, “edges,” “links.” Aim to use just one

**Corrected**

- Page 12, very bottom, typo with “in favour of the new Pop/Rock starts.” Should be “stars”

**Corrected**

- Page 15, very bottom, “For instead” doesn’t seem like the right transition

**For instead is used to introduce an example. I think it is fine.**

Reviewer #2: I would like to that the author for considering the suggestions given by both referees (especially those regarding the temporal aspect). I find the current manuscript much improved. The are some points that I would like to discuss before recommending the manuscript for publication.

1) I am glad that the author modified the measures as suggested and took weights into account. In fact, answering a point previously raised, the author says that the "Degree was replaced by the Weighed degree. Now, the results are more consistent." Was it the same for the other measures? At the moment it is not clear to me if all measures have been changed to their weighted counterparts or just the degree. This is important for consistency and reproducibility.

**Betweenness centrality has been recalculated taking weights into account (see Table 4). This is also made explicit in the text, replacing *betweenness centrality* by *weighted betweenness centrality*.**

**From the first version, PageRank was calculated using weights. A sentence was added to Social network analysis > PageRank: “*It was calculated taking weights into account*.”**

2) Regarding my previous point (2), I obviously agree that musicians are not scientists and a song does not come with a set of references. However, artists with a huge productions have a lot of material available that could be used by other artists when performing covers. In very basic terms, let us assume that an artist wants to record a cover. In the absence of real preferences (tastes or "influence"), the artist would pick a random song from the ones available, and thus would have a higher probability of covering artists with a big production (due to the mass of the production and the age). This implies that --even in the absence of a real influence mechanism-- artists with a lot of songs will end up being more covered than others. I understand the concerns of the author, but, given the central role played by 'influence as a driver for covering' in this manuscript, I believe that this is an important point that has not been addressed and should be (at least) discussed.
In addition, I find the argument 'more songs do not imply more cover versions' not really confirmed here. The correlations coefficients provided "low correlation between the number of songs recorded and covers done (weighted outdegree) (r=.465) and received (weighted indegree) (r=.443)" are not that low to me.

**I think that the discussion about the normalization of covers is very interesting but it goes beyond to the peer-review. Let me go in depth the reasons why the normalization is not suitable in this study:**

**As I have commented to the first reviewer, the information about the number of songs that Allmusic provides is not entirely correct. Allmusic computes the number of different recordings, even these different sessions are about the same song. This introduce a bias in favour of the most popular singers, because they have more published material than others (i.e. Greatest hits, Live discs, hidden sessions, etc.). I think that this variable is unstable and it is not suitable to normalize the covers.**

**Second, the normalization in skewed distributions can produce artefacts with unrealistic results. For example, if the normalization is applied, the top 10 artists will be:**

| **Label** | **songs** | **active_period** | **starting_decade** | **main_genre** | **weighted indegree** | **nor weighted indegree** |
| --- | --- | --- | --- | --- | --- | --- |
| **Franz Gruber** | **2** | **1890s** | **1890s** | **Classical** | **3157** | **1578.5** |
| **Joseph Mohr** | **2** | **1890s** | **1890s** | **Classical** | **3157** | **1578.5** |
| **Emily Laurey** | **1** | **1890s** | **1890s** | **Classical** | **1557** | **1557** |
| **Meister Glee Singers** | **1** | **1890s** | **1890s** | **Vocal** | **983** | **983** |
| **Hollace Shaw** | **1** | **1930s** | **1930s** | **Stage & Screen** | **975** | **975** |
| **Hiram Sherman** | **1** | **1930s** | **1930s** | **Vocal** | **966** | **966** |
| **Gloria Grafton** | **1** | **1930s** | **1930s** | **Stage & Screen** | **944** | **944** |
| **Tally-Ho!** | **1** | **1900s** | **1900s** | **Vocal** | **919** | **919** |
| **Kathryn Crawford** | **1** | **1930s** | **1930s** | **Stage & Screen** | **770** | **770** |

**The result shows that these authors have a very high impact due to by only one or two songs. The first three are due to a popular Christmas song. The other one are unknown singers with only a punctual popular hit. The question is then, are these authors the most important in the contemporary popular music? I do not think so. Then, the normalization in this case provide poor results.**

**You say: “In the absence of real preferences (tastes or "influence"), the artist would pick a random song from the ones available, and thus would have a higher probability of covering artists with a big production (due to the mass of the production and the age).”**

**Correct. A random selection means that every song has the same likelihood to be elected, then the distribution will be normal or Gaussian. But the distribution is skewed, then it should be a non-random selection criterion and there is not the same likelihood to be elected. That is, it does not matter how many songs you have if they are not enough goods or interesting, they will not be covered.**

**I have included a footnote to explain why this normalization was not applied:**

**“*Data supplied by Allmusic. Number of musical tracks recorded and released by artist. It may include different recordings of the same song. Due to this and the skewed distribution of cover songs (Figure 1), this data was not used as normalization variable.*”**

Minor points:

When I read 'evolutionary' I think about evolution as a change (like in biology). I thus find its use in some parts of the manuscript a bit our of context, since it reads more like a temporal analysis (how quantities change in time) rather than the analysis of how things develop (by changing into others).

**I have replaced *evolutionary* by *longitudinal***

In Objectives, 'indicators' is repeated.

Just before Fig.2, 'The artists that joint the network'--> joined

**Corrected**

The text regarding Fig. 2 says "From a longitudinal point of view, the growing pattern of the network is not continuous (Figure 2)". I find the sentence 'growing pattern of the network' a bit misleading, since degree distributions are skewed (and the average is not much representative) and the real pattern I assume would be hidden in the fact that new coming nodes without many links pull down the average after the initial growing phase. The author might consider plotting also the number of new nodes/covers in time to have a clearer picture of the growing pattern of the network that generate the curves shown. In addition, I would suggest avoiding the use of 'not continuous' since it might imply the presence of a discontinuity in the curve (which is not the case).

**Table 1 publishes the number of nodes and arcs in each time, so the readers can see the difference between each period. It is normal to assume that there are new coming nodes with a low number of covered songs. This is because precedent artists scarcely cover songs from subsequent artists. However, I do not see that new nodes pull down the average because in spite the new nodes are not covered still, the former ones are indeed being covered by the new nodes, then the average can growth (as it does happen in the 1900-1950s period).**

**I have replaced *continuous* by *constant.***

After Fig. 4, "This fact then suggests that the covering of songs is more and more concentrated in few artists, increasing exponentially their impact over time." how is "exponentially" tested?

***Exponentially* was used as a descriptive adverb. The trend was not tested. I have replace exponentially by *extremely.***

In Fig. 4 and 5 I find difficult to associate artist labels with curves when there are many (for example The Rolling Stones and Bob Dylan in FIg.5). Maybe arrows could help?

**Those figures were edited to introduce arrows between labels and lines.**

Fig. 8 is hard to read since blues colors are only slightly different (I can guess due to their size, but it is not straightforward).

**Figure 8 was replaced by a heat map**

Figure captions seem to include caption titles only. These should be improved by better explaining the variables shown.

**Some captions have been rewritten to detail information about the variables.**
