# Supplementary material for: Cover versions as an impact indicator in popular music: A quantitative network analysis
Source: PLoS One. 2021 Apr 19;16(4):e0250212. doi: 10.1371/journal.pone.0250212 (PMC8055012; doi:10.1371/journal.pone.0250212)
Supplement: Attachment — Submitted filename: Response to Reviewers_2.docx [file pone.0250212.s006.docx]

6. Review Comments to the Author

Please use the space provided to explain your answers to the questions above. You may also include additional comments for the author, including concerns about dual publication, research ethics, or publication ethics. (Please upload your review as an attachment if it exceeds 20,000 characters)

Reviewer #1: The author has addressed most of my concerns. I appreciate the work that was done to respond to both of the reviewers.

Reviewer #2: I would like to that the author again for implementing the suggested modifications, improving in this way the overall presentation of the manuscript. Based on the latest changes, I would recommend publication in PLoS One --even though I am still a bit puzzled by the entire normalisation argument.

In particular, there are two crucial points from the latest answer:

1) When the author writes "the information about the number of songs that Allmusic provides is not entirely correct. Allmusic computes the number of different recordings, even these different sessions are about the same song (...) I think that this variable is unstable and it is not suitable to normalize the covers." it seems to me that the non-suitability of the possessed data is used to justify the appropriateness of the employed methodology. While the possibility of doing something is obviously constrained by the data one can have access to, I think that this should not be used as a motivation --but rather as the best possible strategy given the circumstances.

2) The results with the applied normalisation are, according to the author, *poor*, since internationally acclaimed artists do not appear on the top. Again, I agree that an artist that did one single famous Christmas song should not be considered the most influential one in contemporary popular music. However, I also find a bit pointless the need to have the most famous artists we all know at the top of the ranking, where we expect them to be. If this is the case, why doing the entire analysis in the first place? I am obviously exaggerating here, but I believe that the answer lies somehow in between.

As such, I think that a little discussion on this in the conclusion is needed, and the normalised table could be put in the SI accompanied by a short text on its caveats.

**I have added the Limitation section to discuss the normalization problem in skewed distributions and the imprecise definition of songs by Allmusic. In addition, I have added as Supplementary material a table with the top 10 ranking of artists according to the normalized impact by song and a discussion about this result.**
